# Impact of Poverty on Parent–Child Relationships, Parental Stress, and Parenting Practices

**DOI:** 10.3389/fpubh.2022.849408

**Published:** 2022-04-25

**Authors:** Laurie Long Kwan Ho, William Ho Cheung Li, Ankie Tan Cheung, Yuanhui Luo, Wei Xia, Joyce Oi Kwan Chung

**Affiliations:** ^1^The Nethersole School of Nursing, The Chinese University of Hong Kong, Hong Kong, Hong Kong SAR, China; ^2^Xiangya School of Nursing, Central South University, Changsha, China; ^3^School of Nursing, The Sun Yat-Sen University of Medical Sciences, Guangzhou, China; ^4^School of Nursing, The Hong Kong Polytechnic University, Hong Kong, Hong Kong SAR, China

**Keywords:** poverty, parent–child relationship, parental stress, parenting practices, harsh parenting, preschool children, nurse, primary care

## Abstract

**Objectives:**

To explore the impact of poverty on parent–child relationships, parental stress and parenting practices.

**Design:**

A mixed methods study.

**Sample:**

Four hundred and eighty five Hong Kong Chinese parents who had children aged 3-6 years, and who were from low-income families. Eleven of these parents were randomly selected for individual semi-structured interviews.

**Measurements:**

A sociodemographic questionnaire, the parent–child relationship score, the Parental Stress Scale and the Perceived Parental Aggression Scale.

**Results:**

The parents were found to have an impaired relationship with their children. The findings indicated that employment status, parental stress and harsh parenting were significantly associated with parent–child relationships. The qualitative findings revealed that parents from low-income families encountered a wide range of difficulties, which made these parents more likely to experience parental stress, thereby increasing their tendency to adopt harsh parenting practices that undermined parent–child relationships.

**Conclusion:**

This study sheds light on the associations between parent–child relationships, parental stress and parenting practices in low-income families. These findings will enhance nurses' understanding of the impact of poverty on parent–child relationships, and highlight the need for nurses to ensure that underprivileged parents and their children receive adequate primary care to prevent the development of psychological problems in this vulnerable group.

## Introduction

Parent–child relationships play an important role in the development of children, especially during their preschool years (1, 2). Poverty can place severe strains on parent–child relationships, due to the highly stressful conditions caused by financial problems and material deprivation, thereby generating negative psychological effects and family dysfunction (3, 4). Psychological health is defined as an integral part of health, in which an individual is capable to recognize his/her own abilities and cope with the stresses of life (5, 6). In particular, the quality of parent–child relationships is known to be a crucial determinant of the psychological health and life satisfaction of children from low-income families (7). Lower-quality parent–child relationships jeopardize children's cognitive development and increase their risk of developing behavioral and psychological problems (8). Moreover, a systematic review revealed that preschool children from low-income families were five times more likely to have behavioral problems than other age-group children from such families (9, 10). Preschool children with developmental problems have also been shown to exhibit weaker adaptive abilities in later life than those without such problems, which makes such preschool children more prone to develop severe behavioral and psychological problems during adolescence and adulthood (9, 11, 12).

The family stress model explains the impact of poverty and poor household economic conditions on children and their parents (13). This model also demonstrates how poverty and economic hardship amplify parental stress and hence increase inter-parental conflict, which in turn leads to an increase in the adoption of harsh parenting practices (3, 14). These harsh parenting practices not only impair parent–child relationships (3), but also cause mental and developmental maladjustment in children, including decreased cognitive functioning (15), reduced social competence (16), and impaired psychological health, such as depression and anxiety (17).

Most of the literature in this area has focused on adolescents and has thus failed to recognize the importance of parent–child relationships during preschool years, which is a critical stage in life during which children are at a high risk of developing mental and behavioral problems, especially those who are underprivileged (18). The existing evidence also fails to provide a qualitative understanding of the impact of poverty on parent–child relationships, parental stress and parenting practices. A more comprehensive insights of this topic would assist healthcare professionals to design appropriate interventions for enhancing parent–preschool child relationships in low-income families, and thus ensure that there is adequate support for the prevention of psychological problems in this population. It is also imperative that effective psychological interventions are developed for this subset of low-income families, as they may enhance the health of these families and reduce poverty-related burdens on healthcare systems.

The wealth disparity in Hong Kong is the highest among all developed countries, as indicated by the Gini coefficient (19). This is illustrated by the fact that over the past decade, the number of children aged under 18 years in Hong Kong who were living in poverty has increased by 10,800, to 181,200 (20). In addition, the child poverty rate was 17.8% in 2019, indicating that approximately one in five children in Hong Kong was living in poverty. The increasing number of children living in poverty reveals that a substantial proportion of children are susceptible to the adverse impacts of poverty, including undermined parent-child relationship. In particular, lockdown policies (including school closure) during the COVID-19 pandemic are found to greatly affect the psychological well-being of parents and children from low-income families, owing to confinement of their small homes (e.g., subdivided flats) for long hours and limited space for movement. This could also increase family conflict, which might further undermine the parent-child relationship of low-income families during the pandemic. This study, therefore, aimed to (1) examine the level of parent–child relationships, parental stress and harsh parenting practices in low-income families comprising Hong Kong Chinese parents and their preschool children; (2) investigate the relationships between parent–child relationships, parental stress and harsh parenting in this population; and (3) explore how the lived experiences of and challenges faced by parents in this population affect their parenting practices and their relationships with their children.

## Materials and Methods

### Aims

This study aimed to examine and explore the impact of poverty on parent–child relationships, parental stress and parenting practices.

### Design

We conducted an exploratory study using quantitative and qualitative approaches. The methods and results are reported according to the Good Reporting of a Mixed Methods Study (GRAMMS) checklist.

### Sample

We defined low-income families as families with a monthly income at or below the half-median monthly domestic household income of Hong Kong, or those who received Comprehensive Social Security Assistance (19). Parents from low-income families were recruited if they: (i) had children aged between 3 and 6 years, (ii) were able to speak Cantonese and read Chinese, and (iii) had completed primary school education or above. Parents with identified cognitive and learning problems were excluded, as were children with such problems. Only one parent, either father or mother, from each family, who was the primary caregiver of children, was recruited.

### Data Collection

The recruitment of participants (i.e., parents) was conducted in Sham Shui Po community, which is the Hong Kong district with the highest poverty rate (19). All participants were asked to complete a set of questionnaires. In addition, 11 participants were randomly selected for individual semi-structured interviews, which lasted for approximately 15–25 min and were conducted in a private meeting room. These interviews were audio-taped with participants' permission.

### Measures

#### Socioeconomic and Demographic Characteristics

Participants' socioeconomic and demographic data were collected, comprising their age, sex, marital status, educational level, employment status, whether they were born in Hong Kong, the type of housing they lived in, religious belief and number of children.

#### Parent–Child Relationship

The parent–child relationships of the participants were assessed using a self-reporting method containing the following two items: “How satisfied are you with your parent–child relationship?” and “As a parent, how satisfied are you with yourself?” (21). Each item is rated on a six-point Likert scale from 1 (totally unsatisfactory) to 6 (totally satisfactory). A higher score indicates a better parent–child relationship.

#### The Chinese Version of the Parental Stress Scale

The PSS was developed to measure parents' perception of the parental stress they experienced when raising children (22). The Chinese version of the PSS was translated by Cheung (23) and consists of 17 items that are rated on a six-point Likert scale from 1 (disagree very much) to 6 (agree very much). A higher score represents a higher level of parental stress. The Chinese version of the PSS has demonstrated acceptable psychometric properties (23) and is therefore suitable for use by researchers to assess the parental stress levels of Chinese parents.

#### The Chinese Version of the Perceived Parental Aggression Scale

The Chinese version of the PPAS was used to assess the level of harsh parenting practices, such as parents' level of physical and verbal aggression toward their children. It is one of the subscales of the Parental Acceptance Rejection Questionnaire, which is a parent self-report questionnaire (24). It consists of 15 items that are measured on a five-point Likert scale from 1 (never) to 5 (always). A higher score indicates a higher level of aggression and hostility toward one's children. The psychometric properties of the Chinese version of the PPAS have been empirically investigated and indicate that it has high reliability and validity for assessing the level of harsh parenting practices (24, 25).

### Semi-structured Interview

Participants were randomly selected from the quantitative study, using a computer-generated random number list, and invited to attend individual semi-structured interviews. The interview sample size was determined by data saturation, which was achieved after interviewing 11 participants. The interviews aimed to explore parents' lived experiences and challenges related to poverty, which affect parent–child relationships. All the interviews were conducted by a registered nurse, who was not involved in the quantitative data collection and was trained by a professor who is experienced in qualitative research and childcare so as to gain the competence to communicate with parents. A semi-structured interview guide was developed to explore three major areas: parents' perceptions of being a parent, parents' perceptions of their relationships with their child(ren) and the daily poverty-related challenges experienced by parents that affected parent–child relationships. The interviews began with some broad and open questions, such as: “Can you share something about you and your child(ren)—for example, how would you describe your relationship with your child(ren)?” This was followed by questions related to major areas in the interview guide [e.g., “How does your family income or financial status affect you and your child(ren)'s daily lives?” or “In what ways does your present financial situation affect your relationship with your child(ren)?”]. Nondirective and repetitive supplementary questions (e.g., “Why do you feel like this?” or “Can you give me some examples?”) were used to elicit more detailed information. Different probing techniques were also applied throughout the interviews to evoke more responses and more comprehensive information.

### Ethical Considerations

Ethical approval was obtained from the Institutional Review Board (UW16-250) and written informed consent was obtained from the participants. We ensured that the participants understood that their participation was voluntary, that they could withdraw from the study at any time without any negative consequences, and that the information collected would remain confidential.

### Analytic Strategy

SPSS Statistics for Windows, Version 23.0 (IBM Corp, Armonk NY, United States) was used for quantitative data analysis. Descriptive statistics were used to calculate the frequencies and percentages of socioeconomic and demographic characteristics. Data on parent–child relationships and results of the Chinese versions of the PPAS and PSS were presented as means and standard deviations. Pearson product-moment correlation coefficients were calculated to investigate the relationships between parent–child relationships, PPAS and PSS scores and socioeconomic and demographic characteristics. In addition, a multiple regression model was used to explore factors that may significantly affect parent–child relationships. The parent–child relationship score was set as the dependent variable. The independent variables were selected using the Pearson product-moment correlation coefficients of particular variables, namely age, sex, marital status, employment status, born in Hong Kong, parental stress and harsh parenting.

### Rigor

Colaizzi's (26) descriptive phenomenological data-analysis strategy was used to analyze the qualitative data. To accurately capture the content of the dialogue and physical expressions, all interview recordings were transcribed verbatim into Cantonese. Important quotations relevant to the themes were identified and translated into English.

First, the transcripts were reviewed intensively multiple times to gain a general sense of the constructs or ideas in the content. Then, content relevant to the major areas covered in the interview guide was extracted and labeled as significant statements. These significant statements were used to formulate different meanings about the content. The formulated meanings were then grouped into categories and themes by examining and identifying their similarities. Ultimately, exhaustive descriptions were obtained from the themes.

The quality and rigor of the qualitative study, in terms of its credibility, transferability, dependability, and confirmability, were ensured using several strategies. Triangulation strategies were adopted to enhance credibility, such as by taking field notes throughout the interviews to capture any supplementary nonverbal cues and involving two researchers in the process of data analysis (27). Member-checking was also performed to enhance credibility, by validating results with the participants (27). Moreover, interview privacy was ensured by offering a safe and secure environment for the participants. The participants were assured of confidentiality to encourage them to feel free to express their feelings and ideas honestly. Transferability was demonstrated by identifying similarities to other research findings and was enhanced by using direct quotations of the participants and explicit descriptions of their experiences.

Dependability was achieved by using the technique of stepwise replication (28). This involved two researchers analyzing the data independently, and then comparing their findings to ensure stability and consistency. Additionally, all of the interviews were carried out by the same researcher to maintain consistency. Finally, confirmability was improved by reflecting on the process of data analysis (28), which involved the two researchers recording the procedures of data analysis and periodically reflecting on these procedures to maintain their objectivity. Research team meetings were also held at regular intervals to monitor the data-analysis process and manage any divergence of opinion.

## Results

### Quantitative Analysis

From March 2017 to January 2018, 485 participants were recruited by convenience sampling. The response rate was 91%; 49 participants refused to join the study because of unavailability or no interest in participating. The participants' socioeconomic and demographic characteristics are shown in Table 1. The participants had a mean age of 35.6 years and most (91.5%) were mothers. A majority of the participants were married (80%), had a secondary educational level (76.5%), were unemployed (78.1%) and did not have a religious belief (85.2%). Most were living in subdivided flats (42.5%) and public rental housing (38.8%). Two hundred and twenty-three participants (46%) had two children and 195 (40.2%) had one child.

**Table 1:** Socioeconomic and demographic characteristics of participants (*N* = 485).

	**No. (%)**
Age, mean (SD), year	35.55 (6.33)
**Sex**	
Male	41 (8.5)
Female	444 (91.5)
**Marital status**	
Married	388 (80.0)
Divorced/ separated	97 (20.0)
**Educational level**	
Primary	30 (6.2)
Lower secondary	184 (37.9)
Higher secondary	187 (38.6)
Tertiary	84 (17.3)
**Employment status**	
Employed	106 (21.9)
Unemployed	379 (78.1)
**Born in Hong Kong**	
Yes	302 (62.3)
No	183 (37.7)
**Types of housing**	
Subdivided flats	206 (42.5)
Public rental housing	188 (38.8)
Private rental housing	87 (17.9)
Private housing (owner)	4 (0.8)
**Religious belief**	
Yes	72 (14.8)
No	413 (85.2)
**Numbers of children**	
One	195 (40.2)
Two	223 (46.0)
Three	49 (10.1)
Four	18 (3.7)

The mean parent–child relationship, PPAS and PSS scores are shown in Table 2. The results showed that the mean parent–child relationship score of the participants was slightly higher than the midpoint of the scoring ranges (range: 1–12; mean = 7.47; SD = 2.25), implying that the participants have an impaired relationship with their children. The results also revealed that the participants had rather high levels of parental stress (range: 17–102; mean = 69.86; SD = 11.12), indicating that they experienced a significantly high level of stress when raising their children. Moreover, the mean score of harsh parenting practices was slightly above the midpoint of the scoring ranges (range: 15–75; mean = 39.49; SD = 10.79), showing that the participants might have a slight tendency to adopt harsh parenting practices.

**Table 2:** The mean score for parent-child relationship, PPAS and PSS (*N* = 485).

	**Mean (SD)**
Parent-child relationship	7.47 (2.25)
Parental stress	69.86 (11.12)
Harsh parenting	39.49 (10.79)

The relationships between parent–child relationships, PPAS and PSS scores, and socioeconomic and demographic characteristics are shown in Table 3. The results revealed that parent–child relationships were negatively associated with parental stress and harsh parenting. In addition, sex, marital status, employment status and being born in Hong Kong were also found to be significantly associated with parent–child relationships. Table 4 summarizes the results of a multiple regression analysis of variables that may predict participants' relationships with their children. Age and sex were entered at Step 1, explaining 4% of the variance in parent–child relationships. In Step 2, the other variables related to parent–child relationships were entered, namely marital status, employment status and born in Hong Kong. The variance explained by the model at Step 2 was 15% of the variance in parent–child relationships. After the entry of the variables of parental stress and harsh parenting at Step 3, the overall models explained 55% of the variance. After controlling for the possible effects of age, sex, marital status, employment status and born in Hong Kong, the coefficient of determination (*R*^2^) value changed by 0.40, indicating that parental stress and harsh parenting explained an additional 40% of the variance in parent–child relationships. Notably, after all variables were entered into the model, employment status, parental stress and harsh parenting were identified as factors that were significantly associated with parent–child relationships. Parental stress had the highest standardized coefficient (β = −0.67, *p* < 0.001), followed by harsh parenting (β = −0.12, *p* = 0.01) and employment status (β = 0.10, *p* = 0.03).

**Table 3:** The intercorrelation coefficients among parent–child relationships, PPAS and PSS scores, and socioeconomic and demographic characteristics.

	**A**	**B**	**C**	**D**	**E**	**F**	**G**	**H**	**I**	**J**	**K**	**L**
Age (A)	1											
Sex (B)	−0.16^*^	1										
Marital status (C)	−0.17^*^	0.09	1									
Educational level (D)	−0.10	−0.18^*^	−0.15^*^	1								
Employment status (E)	0.02	−0.27^*^	−0.14^*^	0.10	1							
Born in Hong Kong (F)	−0.06	−0.02	−0.13^*^	0.09	0.03	1						
Types of housing (G)	−0.17^*^	−0.08	−0.15^*^	0.05	0.22^*^	−0.08	1					
Religious belief (H)	0.10	0.04	0.17^*^	0.06	−0.01	−0.08	0.13	1				
Numbers of children (I)	−0.18^*^	0.02	0.18^*^	0.03	−0.04	−0.02	0.00	–.03	1			
Parent-child relationship (J)	−0.08	−0.16^*^	−0.23^*^	0.11	0.23^*^	−0.18^*^	0.13	–.03	0.04	1		
Parental stress (K)	0.11	0.16^*^	0.22^*^	−0.13	−0.16^*^	0.29^*^	−0.15^*^	.02	0.06	−0.71^*^	1	
Harsh parenting (L)	0.13	0.14^*^	0.19^*^	−0.11	−0.11	0.06	−0.08	–.03	−0.04	−0.27^*^	0.24^*^	1

* *Significant at p < 0.05*.

**Table 4:** Multiple regression analysis for variables affecting parent-child relationship.

	**Step 1**			**Step 2**			**Step 3**		
	**B^†^**	**SE B^‡^**	**β^§^**	**B**	**SE B**	**β**	**B**	**SE B**	**β**
**Variables**									
Age	−0.04	0.02	−0.11	−0.03	0.02	−0.08	0.00	0.02	0.01
Sex	−1.50	0.54	−0.19^*^	−0.88	0.54	−0.11	−0.09	0.40	−0.01
Marital status				−0.72	0.24	−0.20^*^	−0.14	0.18	−0.04
Employment status				1.00	0.36	0.19^*^	0.57	0.26	0.10^*^
Born in Hong Kong				−1.45	0.43	−0.21^*^	0.14	0.34	0.02
Parental stress							−0.14	0.01	−0.67^*^
Harsh parenting							−0.03	0.01	−0.12^*^
R^2^	0.04			0.15			0.55		
Adjusted R^2^	0.03			0.13			0.54		
R^2^ change = 0.40									

† *Unstandardised coefficients;*

‡ *Standard error of unstandardized coefficients;*

§ *Standardized coefficient*.

* *Significant at p < 0.05*.

### Qualitative Analysis

Eleven parents were recruited for the individual semi-structured interviews. Their mean age was 34.3 years (SD = 3.3). Most were mothers (81.8%) and housewives (63.6%), had received secondary education (90.9%), and born in Hong Kong (63.6%). Four main themes were identified from the qualitative data, and each theme was further divided into subthemes. A summary of themes and subthemes is presented in Table 5.

**Table 5:** Themes and subthemes from the semi-structured interviews.

**Themes**	**Subthemes**
1. Poverty affects parents' perceptions of their parental role	1.1 Effects on the role of mothers and fathers
	1.2 Overwhelmed by their parental role due to their financial situation
2. Financial pressure undermines parent–child relationships	2.1 Long working hours
	2.2 Material deprivation
3. Poverty as a key barrier to building up social networks	3.1 Difficulty in building up social networks due to having a lower financial status than other parents
	3.2 Mainland China immigrants experienced difficulty in integrating into their communities
4. Refusal to seek social support	

### Theme 1: Poverty Affects Parents' Perceptions of Their Parental Role

The theme “poverty affects parents' perceptions of their parental role” emerged in the data for 10 participants (90.9%). Two subthemes were identified.

#### Effects on the Role of Mothers and Fathers

The findings showed that the division of the workload in a family has different effects on parent–child relationships. A majority of the participants stated that the mother in their families was primarily responsible for rearing a child/children at home and that the fathers worked away from home on most of the day. This resulted in the fathers seldom being involved in parenting, as they spent little time with their children; thus, most fathers did not have a close relationship with their children.

“I take my son to the school, cook him meals, and teach him to do homework…. My husband does not have time to play with him… He is fully occupied by his work.” Parent C, female, aged 34.

“Her dad's job involves shift work. Sometimes he and our daughter don't meet each other for a week.” Parent H, female, aged 37.

#### Overwhelmed by Their Parental Role Due to Their Financial Situation

Some of the participants felt overwhelmed by their roles as parents, especially if they were single parents. A majority of the participants agreed that they did not know how to get along well with their children, and sometimes experienced conflict with them. Most of the participants said that poverty put great pressure on them, and they admitted that this sometimes made them emotionally upset, leading to them exhibiting negative emotions in interactions with their children.

“I hope I can give my son the best of everything. However, this makes me feel great pressure because actually, I can give him nothing.” Parent B, female, aged 31.

### Theme 2: Financial Pressure Undermines Parent–Child Relationships

The theme of “financial pressures undermines parent–child relationships” was evident in the comments of 11 participants (100%), and two subthemes were identified.

#### Long Working Hours

Most participants mentioned that because of their low educational levels, they or their partners had to work for over 10 h per day and were only paid the Hong Kong statutory minimum wage (i.e., HK$34.5 per hour). Their relationship with their children was greatly affected by these long working hours.

“My working hours are very long, so I do not have much time to communicate with my daughter. But you know, earning money is very important. Sometimes I will ask her grandma to take care of her.” Parent G, male, aged 39.

#### Material Deprivation

Most of the participants stated that they felt stressed when their children were growing up and starting to make comparisons with their classmates. Some participants mentioned that their children wanted them to buy certain items, such as toys or stationery, which were similar to those of their classmates. However, most of the participants considered that these items were dispensable and that they could not afford such unnecessary expenditure. When they rejected their children's requests for such items, they reported that their children reacted angrily, and appeared to believe that the participants did not love them because the participants did not satisfy their wants, unlike other parents who satisfied the wants of their children. Although these participants understood that their children were too young to understand their family's financial situation, some of the participants nevertheless found it difficult to hide their negative response to their children's anger and hence expressed negative emotions in interactions with their children.

“I admit that I treat my son too harshly sometimes, especially when I refuse to buy him toys. But it is understandable, right? We are poor, so we cannot hope for too much in our lives. I hope he can understand this when he is grown up.” Parent A, female, aged 33.

“My son always thinks that I don't love him because I don't buy him toys, as other parents do for their children. However, our income only covers our daily expenditure. How can I have extra money to buy him toys?” Parent D, female, aged 36.

### Theme 3: Poverty as a Key Barrier to Building Up Social Networks

The theme “poverty as a key barrier to building up social networks” was reported by nine participants (81.8%) and two subthemes were identified.

#### Difficulty in Building Up Social Networks Due to Having a Lower Financial Status Than Other Parents

A majority of the participants mentioned that it was very common to be invited to join social chat groups with other parents on social media platforms (e.g., WhatsApp or WeChat). Some of the participants described other parents as “monster parents” and stated that they felt pressure when listening to other parents describing their parenting practices. The participants stated that other parents always shared the schedules and content of their children's extra-curricular activities or tutorial classes and asked the participants whether their children wanted to join in these activities. The participants understood that their children could benefit by joining in such activities but stated that these activities were so expensive that they could never afford them. In addition, most of the participants said that they minimized daily expenditures by seldom taking their children to activities. Some of the participants were too ashamed to admit to other parents that they were poor, and instead lied, saying that their children did not have time to join these activities. A few of them even chose to quit chat groups. Single parents also mentioned that they never joined social groups, because they did not want to disclose their family situation to others.

“I don't know what other parents think of me because I said “no” every time they invited me and my son to join some activities. Those activities are too expensive for us to afford.” Parent F, female, aged 30.

“In Chinese, we have the saying “*family shames must not be spread abroad.”* Poverty is not a good thing, and I don't want other parents to have any prejudiced viewpoints on me or my daughter. It's best that we keep a distance from other parents.” Parent I, female, aged 39.

“I tried to join their social groups, but I found that our disparities become apparent when we interacted with the rich people in these groups. I felt extra pressure when they asked me to allow my son to join some activities with their children.” Parent H, female, aged 37.

#### Mainland China Immigrants Experienced Difficulty in Integrating Into Their Communities

A few participants reported experiencing difficulties in integrating into their communities, as they were immigrants from mainland China. They felt stressed and required more time to adjust their lifestyles to adapt to the crowded living environment and fast-paced lifestyles of Hong Kong. In addition, the higher living standards of Hong Kong compared with mainland China increased their financial pressure. They stated that since immigrating from mainland China, they spent less time engaging with their children.

“They (other parents) seem to not want to interact with mainland people. Maybe [this is because] they think we are poor and come from a rural area. So, I don't want to start a conversation with them, either.” Parent J, female, aged 30.

### Theme 4: Refusal to Seek Social Support

The fourth theme “refusal to seek social support” emerged in the interviews with seven participants (63.6%). These parents stated that their lives were hard due to financial pressure and a lack of support. However, they felt too ashamed to seek help from their families or the community.

“People think that all mainland China immigrants only know how to ask for money from the government. I am too ashamed to beg for help from others. I feel uncomfortable if I receive help from others, because I feel that I should be able to cope with my current situation.” Parent K, female, aged 33.

“I don't like to seek help. People should gossip about why I divorced. People judge us all the time, especially our family is poor and incomplete.” Parent E, male, aged 35.

## Discussion

This study addressed an understudied area: the impact of poverty on parent–child relationships, parental stress and parenting practices. Since there are no universal cut-off points for these measures, we compared the findings of this study with the baseline data of a previous Hong Kong study investigating the effectiveness of a parental training program in preparing children for their transition to the primary school that used the same measurement scales (21). Compared with parents experiencing a stressful transition, parents in this study showed a lower mean score in parent–child relationships (7.47 vs. 8.31) and higher mean levels of parental stress (69.86 vs. 67.54) and harsh parenting practices (39.49 vs. 35.61). These findings show that poverty has an additional negative impact on parent–child relationships.

The results presented here may not be comparable with those of Western studies, owing to differences in the use of measurement tools between Western and Eastern studies. However, the results are consistent with previous Western study findings that parents from low-income families experienced high parental stress and tended to exhibit harsh parenting practices, thereby damaging parent–child relationships (29, 30).

This study sheds new light on the relationships between socioeconomic and demographic characteristics, parent–child relationships, and parental stress and harsh parenting in low-income families. Specifically, our findings indicate that parental stress and harsh parenting are negatively associated with parent–child relationships. In addition, the role of fathers, single parenthood, unemployment and being a mainland China immigrant is significantly associated with a lower mean parent–child relationship. These findings may be attributable to the fact that these socioeconomic and demographic characteristics could influence the choice of parenting style, and their perceptions of parent-child relationships/children's psychological health. The result of a multiple regression model revealed that employment status, parental stress and harsh parenting are statistically significant contributors to parent–child relationships in low-income families with preschool children. This is additional evidence that employment status, which indirectly reflects household income, is a factor that is significantly associated with parent–child relationships in low-income families. Moreover, parental stress accounted for the largest proportion of variance within parent–child relationship scores, with a β coefficient of −0.67.

The qualitative information in this study provides new insights into the lived experiences of and challenges faced by the participants, and the effects of these on parent–child relationships. It suggests that there are several reasons why poverty affects parent–child relationships in low-income families. For example, as economic pressure depletes an individual's capacity, low-income families might experience more intra-family conflict, resulting in parents being more likely to adopt harsh parenting practices and hence undermine parent–child relationships. Moreover, the long working hours in Hong Kong might decrease the quality of parent–child interactions and thus hinder parent–child relationships, especially father–child relationships. These outcomes illustrate how the mental health and quality of life of children in low-income families might be adversely affected.

Another interesting finding was that parents from low-income families—especially those from single-parent or mainland China immigrant families—were very reluctant to seek community support and build social networks with other parents. These results reflected the influence of a philosophical doctrine of Chinese culture that may lead Chinese people to believe that their fates are controlled by an external and unchangeable force (31). Moreover, Chinese people believe that family shame must not be spread abroad, and may therefore tend to “do not air your dirty laundry in public.” These cultural factors influence parents' choice of coping strategies, and thus, the coping strategies used by parents from low-income families may make them reluctant to seek help. This will make such parents more likely to experience parental stress and hence lead to impaired parent–child relationships. A lack of social support also jeopardizes the social development of children (32).

Additionally, our findings showed that single parents from low-income families might experience more parental stress. This is understandable, as single parents might suffer more financial pressure than dual parents, resulting in single parents experiencing a higher level of parental stress, more severe depressive symptoms and a lower level of self-esteem. Therefore, it could be concluded that single-parent low-income families are at a higher risk of lower quality parent–child relationships than dual-parent low-income families. Furthermore, another Hong Kong study found that there is inadequate community support in Hong Kong for single-parent low-income families (33); such a lack of psychosocial support might imperil the mental health of this population, leading to an increased level of parental stress and harsh parenting practices. This would decrease the quality of parent–child relationships, thereby deleteriously affecting the psychological well-being of children. Notably, the qualitative interviews found that some of the single-parent low-income families were ashamed to seek help, implying that additional attention from community nurses/social workers is warranted for identifying those who need further support. They could collaborate with non-governmental organizations which provide support/services for low-income families to reach this subgroup.

### Limitations

This study has some limitations. First, the generalizability of our findings may be limited, as this study used a convenience sample. Second, the cross-sectional study design only revealed associations and did not identify causal relationships between outcome variables. Third, proxy reports were used because of the cognitive incompetence of preschool children.

## Conclusion

This study addressed a literature gap by investigating the impact of poverty on parent–child relationships, parental stress and parenting practices in low-income families comprising Hong Kong Chinese parents and their preschool children. The findings show that healthcare professionals must design and evaluate appropriate interventions to enhance relationships between parents and their preschool children in such low-income families, particularly in single-parent families, to promote the mental health of this vulnerable population.

## Relevance to Public Health

This was an original study that helped to clarify the impact of poverty on the relationships between parents and their preschool children, which is an underexplored area. This study adopted a mixed methods approach by using quantitative and qualitative methods to address the research question and to better understand the lived experiences of those affected by poverty. The results of this study provide a comprehensive picture of how poverty affects parent–child relationships in low-income families. In addition, participants were conducted in Sham Shui Po community, which is the Hong Kong district with the highest poverty rate, and it is conservatively estimated that this study included approximately 40% of low-income families having preschool children in this district (34). The sample size, therefore, can be considered to be a representative sample of parents from low-income families in this district.

The findings of this study have important implications for public health nursing, as they will enhance nurses' understanding of the effect of poverty on parent–child relationships, parental stress and parenting practices in low-income families. This will allow nurses to design and implement appropriate interventions in the community to promote the psychological well-being and the healthy development of children and their parents. Few such interventions and resources are available for underprivileged Hong Kong parents and their children (35, 36). Given this reality, this study shows that nurses should highlight the primary care needs of this vulnerable group and raise public awareness of the negative effects of poverty. In particular, this study revealed that single-parent low-income families are more susceptible to lower quality parent–child relationships, and thus, more consideration should be given to this group of families during the allocation of healthcare resources and the design of future public-welfare policies. Future studies could build on the insights of this study by investigating the impact of poverty on the parent-child relationships among different age ranges of children and/or adolescents; as well as the relationship between parent-child relationship and different parenting styles (e.g., authoritative parenting style).

## Data Availability Statement

The raw data supporting the conclusions of this article will be made available by the authors, without undue reservation.

## Ethics Statement

The studies involving human participants were reviewed and approved by Institutional Review Board of the University of Hong Kong/Hospital Authority Hong Kong West Cluster (HKU/HA HKW IRB). The patients/participants provided their written informed consent to participate in this study.

## Author Contributions

LLKH initiated the study design, analyzed and interpreted the data, and drafted the manuscript. WHCL and ATC contributed to the study design, analyzed and interpreted the data, and critically revised the manuscript for important intellectual content. YL, WX, and JOKC provided practical and research knowledge on study implementation. All authors have read and approved the final version of the manuscript.

## Funding

This study was supported by the Music Children Foundation. The funding source had no involvement in study design; in the collection, analysis, and interpretation of data; in the writing of the report; and in the decision to submit the article for publication.

## Conflict of Interest

The authors declare that the research was conducted in the absence of any commercial or financial relationships that could be construed as a potential conflict of interest. The reviewer AS-WC declared a shared affiliation with the author JC to the handling editor at the time of review.

## Publisher's Note

All claims expressed in this article are solely those of the authors and do not necessarily represent those of their affiliated organizations, or those of the publisher, the editors and the reviewers. Any product that may be evaluated in this article, or claim that may be made by its manufacturer, is not guaranteed or endorsed by the publisher.
